# Fungi without borders: cross-ecosystem decomposition of riparian leaf litter

**DOI:** 10.1093/ismeco/ycag187

**Published:** 2026-07-07

**Authors:** Callie Ochs, Michaela Hayer, Raina Fitzpatrick, Shannon Trottier, Egbert Schwartz, Bruce Hungate, Jane Marks

**Affiliations:** Center for Ecosystem Science and Society, Department of Biological Sciences, Northern Arizona University, Flagstaff, AZ 86011, United States; Center for Ecosystem Science and Society, Department of Biological Sciences, Northern Arizona University, Flagstaff, AZ 86011, United States; Center for Ecosystem Science and Society, Department of Biological Sciences, Northern Arizona University, Flagstaff, AZ 86011, United States; Center for Ecosystem Science and Society, Department of Biological Sciences, Northern Arizona University, Flagstaff, AZ 86011, United States; Center for Ecosystem Science and Society, Department of Biological Sciences, Northern Arizona University, Flagstaff, AZ 86011, United States; Center for Ecosystem Science and Society, Department of Biological Sciences, Northern Arizona University, Flagstaff, AZ 86011, United States; Center for Ecosystem Science and Society, Department of Biological Sciences, Northern Arizona University, Flagstaff, AZ 86011, United States

**Keywords:** leaf litter, decomposition, phyllosphere, aquatic, terrestrial, qSIP, fungi

## Abstract

Fungi are key drivers of leaf litter decomposition in riparian zones, with communities made up of phyllosphere fungi (fungi present on leaves prior to senescence), and fungi that colonize from the environment. While phyllosphere fungi dominate early decomposition in both submerged and terrestrial litter, their contribution to fungal community structure and growth across these environments remains unclear. We used quantitative stable isotope probing to quantify taxon-specific growth rates of phyllosphere fungi and fungi colonizing from the environment, and to differentiate growing fungi from dormant or dead fungi. We quantified the growth rates of phyllosphere and colonizing fungi on two leaf types in aquatic and terrestrial environments over three time points. Growing fungal communities showed substantial overlap between aquatic and terrestrial environments, although this overlap decreased over time. Phyllosphere fungi were more likely to be shared between environments than colonizing fungi. Leaf type exerted a stronger influence on community composition than the environment. Phyllosphere fungi dominated relative abundance and growth during the study, and all fungi grew consistently faster on terrestrial litter. These findings demonstrate the central role of phyllosphere fungi in fungal communities across both aquatic and terrestrial ecosystems, as well as the importance of leaf species.

## Introduction

Fungal communities are key drivers of decomposition globally [1], and understanding the assembly processes and functional roles of these fungi allow us to better understand ecosystem functioning [2, 3]. Riparian leaf litter decomposition provides an ideal model to investigate these dynamics, as riparian zones serve as convergence points for diverse fungal communities, including those from aquatic, soil, and phyllosphere environments [4]. Phyllosphere fungi inhabit living leaves or needles before leaf senescence and decomposition [5]. Some phyllosphere fungi contribute to leaf decomposition in both terrestrial and aquatic environments [6–9] and often benefit from priority effects, where the earliest colonizers get an advantage [10, 11]. Since riparian leaves could fall into either an aquatic or terrestrial environment, studying phyllosphere fungi on riparian leaf litter offers a unique opportunity to assess fungal specialization and the extent of priority effects across differing environments. Additionally, riparian ecosystems host diverse leaf species, allowing us to evaluate whether leaf identity or environmental context (terrestrial vs. aquatic) exerts a stronger influence on fungal community assembly and function.

Aquatic and terrestrial environments impose distinct selective pressures on fungal decomposers. In aquatic systems, leaching and litter fragmentation are consistently higher than in terrestrial environments due to continuous water flow and greater densities of macroinvertebrate decomposers [12–14]. This rapid leaching and fragmentation quickly remove soluble constituents and can dampen chemical differences among leaf types. Aquatic hyphomycetes still deploy broad suites of hydrolytic enzymes (e.g. for pectin, hemicellulose, and cellulose), but lignin degradation on submerged leaves appears comparatively limited (e.g. low peroxidase activity), so lignin-rich residues may persist as other fractions are lost [15]. While water availability is constant in streams, aquatic fungi are often limited by oxygen availability [16, 17]. In contrast, terrestrial fungi are frequently water limited, but have a wider range of dispersal options [15]. Terrestrial fungal communities are more likely to contain basidiomycetes, which can disperse via mycelial cords and hyphae [18]. This hyphal dispersal gives terrestrial basidiomycetes a competitive advantage over bacteria by allowing them to bridge air gaps in search of water and nutrients—an advantage less relevant in aquatic systems [19]. Despite these contrasts between aquatic and terrestrial selective pressures, phyllosphere fungi thrive in both environments, particularly during early decomposition [8, 20, 21]. This study uses taxon-specific relative growth rates to compare fungal activity, whether phyllosphere or colonizing, on terrestrial and aquatic litter.

Another factor that can influence fungal community structure, biomass, and growth is leaf species and leaf quality. This study compared fungal communities on a labile leaf type, Fremont cottonwood (*Populus fremontii*), and a recalcitrant leaf type, Arizona sycamore (*Platanus wrightii*). Fungal taxa on recalcitrant leaves are more often leaf-species specific, as they must degrade complex compounds, like cellulose and lignin [22]. In contrast, labile leaves tend to be dominated by bacteria, rather than fungi [23], but also support higher microbial biomass overall, and a greater abundance of generalist fungi [24]. This means that recalcitrant and labile leaves can be expected to have significantly different microbial communities. Leaching is also a strong differentiator between the two leaf types; cottonwood leaves leach significantly more carbon and nitrogen than sycamore leaves [25, 26], meaning that there is less organic matter available to microorganisms on the leaf and more organic matter available for microorganisms in the water column.

Fungal succession occurs during leaf litter decomposition, where one group of fungi replaces another [27–29]. Phyllosphere fungi are dominant in the early stages of decomposition but are outcompeted by colonizing fungi over time [30–32]. Terrestrial decomposition is driven by a taxonomic shift from ascomycetes to basidiomycetes [28], and a functional shift from hemicellulose decomposition to cellulose and lignin decomposition [31]. Aquatic fungal decomposer succession is driven by the relative frequencies of taxa belonging to the functional group known as aquatic hyphomycetes, which are aquatic specialists rather than fungi coming in with the leaf [33, 34]. Although species turnover of fungal communities has been characterized over periods of leaf decomposition, the drivers of these community changes remain unclear. This study aims to determine how phyllosphere fungi drive fungal community assembly in aquatic and terrestrial environments, on labile and recalcitrant leaves, over time.

Traditional mycological frameworks have separated aquatic and terrestrial fungi using morphological tools [35], but next-generation sequencing (NGS) has revealed phylogenetic overlap between these groups [36] and frequent fungal movement between terrestrial plant tissues and aquatic habitats [37, 38]. What NGS has not been able to show is how this fungal movement from one environment to another contributes to the fungal community function in the new environment. To quantify the growth rates of phyllosphere fungi within aquatic and terrestrial decomposer communities, this study uses quantitative stable isotope probing (qSIP) to differentiate between growing and non-growing taxa and provide taxon-specific metrics of growth. This method uses isotopically labeled water (H₂^18^O) to incorporate heavy oxygen into newly synthesized DNA, increasing its density [39]. Taxon-specific shifts in DNA density between isotopically labeled samples and unlabeled samples indicate taxon-specific relative growth rates *in situ,* with minimal disturbance to the natural fungal community [40]. By using qSIP, we can compare growth rates of phyllosphere and non-phyllosphere fungi across aquatic and terrestrial environments, and between labile and recalcitrant leaf types, and determine which fungal taxa are growing, rather than just being present but dormant, in these treatment combinations.

This study addresses the following questions:

1. Which factor—leaf environment (terrestrial vs. aquatic) or leaf species—has a greater influence on total fungal community structure?
2. How does fungal community growth differ between aquatic and terrestrial environments?
3. How does phyllosphere fungi abundance and growth change over time?

We the expect environment to be a stronger determinant of fungal communities than leaf type because different traits are advantageous in aquatic and terrestrial environments. We expect this to hold for both phyllosphere and colonizing fungi but for it to be most pronounced in colonizing fungi. We predict that phyllosphere fungi will show stronger affinity to leaf type than colonizing fungi due to initial differences in species composition and priority effects. We expect that phyllosphere fungi will dominate fungal growth and abundance during early decomposition in both environments, but that their relative abundance and growth rates will decline over time. Given the accelerated decomposition process in aquatic environments, we predict that fungal growth will be faster in water than on land.

## Materials and methods

Our field site is Beaver Creek, AZ, USA (34°40′09.7′′N 111°42′51.1′′W), a low order forested stream with vegetation typical for a riparian zone in this area [41]. Leaves were collected from Arizona sycamore (*P. wrightii*) and Fremont cottonwood (*P. fremontii*) by placing netting over branches before leaf fall in November, then collecting senesced leaves from this netting after leaf fall in early January. We pooled each leaf type, and then cut enough leaf disks for five 0.25 g DNA extractions of each leaf type to characterize phyllosphere community. If an amplicon sequence variant (ASV) was found in at least two replicates of these senesced leaves pre-decomposition, it was assigned to the phyllosphere group. It is worth noting that there may have been low-abundance phyllosphere fungi that were not detected at this step. We placed 4 g of each leaf type in a fine mesh bag, then attached these bags to rebar. We placed half of the leaf bags in the stream along a 100 m long stretch of pool for 7, 14, and 28 days, and half of the leaf packs on the river bank under a small amount of leaf litter for 7, 28, and 135 days. Terrestrial litter was left to decompose longer since it decomposes significantly slower than aquatic litter [42]. Because of the limited spatial scale of this study site, care should be taken applying these results to more generalized sites.

We prepared 120 falcon tubes for field deployment, 60 for the aquatic incubation and 60 for the terrestrial incubation. To prepare for the terrestrial incubation, we collected soil from alongside the river and partitioned 4 g of soil into sixty 50 ml falcon tubes. Tubes were placed in a drying oven at 60°C to remove soil moisture. To prepare for the aquatic incubation, we collected stream water and put 2 ml of stream water in sixty 15 ml falcon tubes. These tubes were allowed to completely dry down in a drying oven at 60°C to leave only stream water solutes, then either 2 ml of 98 at% ^18^O water or 2 ml of distilled water was added back to the tubes.

When leaf packs were removed from the stream at their respective time points, 110 6 mm disks were cut out of the leaf litter from each pack in the field. We randomly assigned 50 leaf disks to the H_2_^18^O tubes and 50 leaf disks were randomly assigned to the natural abundance water tubes, while 10 leaf disks were kept back to measure mass of leaf disks removed. For the terrestrial samples, leaves were placed on top of soil in the previously prepared falcon tubes, then 1 ml of either H_2_^18^O or natural abundance water was added in small droplets from a syringe to simulate a rain event. Stream water tubes were left to incubate in a flow-through bucket in the stream for 7 days, and terrestrial sample were partially buried in the river bank for 7 days. All tubes were placed immediately back in their respective environments for minimal disturbance. Since fungal growth rates were calculated based on this falcon tube incubation, it is worth noting that our results do not represent natural conditions. Tubes were transported back to the lab on ice and frozen at −80°C until ready for lab work.

DNA was processed and sequenced according to Hayer et al., 2022. Briefly, DNA was extracted using Qiagen DNeasy Soil Pro kit and quantified using a Qubit fluorometer. DNA was then separated by density using an Optima Max benchtop ultracentrifuge at 50 000 rpm at 18°C for 72, and DNA density was determined with internal density standards [43]. Density fractions were then quantified using quantitative PCR (qPCR) with 18S rRNA primers. Standard curves for this 18S qPCR were generated using genomic *Saccharomyces cereviciae* DNA (ATTC 204508D-5). The 10 μL reactions contained 0.25 μM of the primers FR1/FF390 [44], 1× Forget me not PCR Master Mix (Biotium), and 2 mM MgCl2. The assay was performed on a CFX 384 (Bio-Rad, Hercules, CA), using a program of 95°C for 2 min followed by 40 cycles of 95°C for 30 s, 50°C for 30 s, and 72°C for 45 s. Fungal gene copy numbers were calculated using a regression equation for each assay relating the Ct value to the known number of copies in the standards. DNA was amplified using 5.8S-Fun and ITS4-Fun primers [45] and sequenced on an Illumina MiSeq according to the manufacturer protocol.

**Figure 1:**
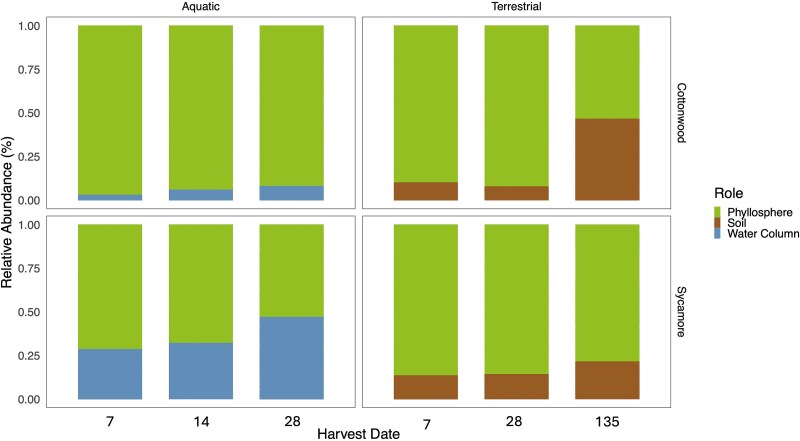
Relative abundance of phyllosphere fungi compared to fungi colonizing from the water column or soil.

ASVs were assigned using the DADA2 [46] plugin in Qiime2 (version 2023.9) [47]. This pipeline yielded 12 118 860 sequence reads assigned to 10 150 ASVs. No rarefaction was used. Taxonomy was assigned using the UNITE full Eukaryotic database at 97% similarity (version 92023-07-18) [48]. Excess atom fraction (EAF) of each taxon was determined using the qSIP2 package [49] in R (version 4.2.2) in RStudio [50]. Taxa that were not found in 2 of the 5 replicates and 3 of the 23 fractions were excluded from the analysis. We used EAF to calculate relative growth rate using the formula: relative growth rate (RGR) = 18O-EAFtaxon/[(0.6)*7 days] [51]. Relative growth rate is given in units of new ITS copies_taxon_/(total ITS copies_taxon_ * day), and relative abundance is given in units of total ITS copies_taxon_/total ITS copies_sample_. To calculate new ITS copies per gram leaf litter per day, which we are using as a proxy for taxon-specific absolute growth rate, we used the formula: (ng DNA/g leaf litter) * (#18S rRNA gene copies/ng DNA) * Relative Growth Rate_taxon_ * Relative abundance_taxon_. For the purposes of this unit (new ITS copies per gram leaf litter per day), it was assumed that the number of 18S copies and the number of ITS copies have a linear correlation, since both correlate linearly to fungal biomass [52, 53]. However, this linear relationship is not well established, and so new ITS copies per gram leaf litter should only be used as an estimate of absolute taxon-specific fungal growth rate. For more quantitatively rigorous measures, refer to relative taxon-specific growth rate and relative abundance independently (Fig. 1 and Fig. S1).

A series of principle coordinate analyses (PCoAs) were carried out on relative abundance data using the Bray–Curtis metric of dissimilarity. A PERMANOVA with Bray–Curtis dissimilarity was used to test the differences in fungal community between leaf environment (aquatic vs. terrestrial) and leaf species (cottonwood vs. sycamore), using the vegan function adonis2 (vegan: version 2.6-4) [54]. Both the PCoA visualizations and PERMANOVA were performed based on code provided in Buckley et al., 2021 [55]. Differences in phyllosphere relative abundance at each harvest date were evaluated using a series of logistic regression models for each leaf type × environment combination. These regressions used a generalized linear model with a binomial error distribution implemented in R using the glm function in the stats package. Harvest was included as the only predictor.

## Results

Decomposition was faster in the stream than on soil and faster for cottonwood than sycamore. By the last aquatic timepoint, day 28, cottonwood leaves in the stream lost 47%–49% of their mass, and sycamore leaves in the stream lost 14%–19% of their mass (Fig. S2). In contrast, by day 28, cottonwood leaves on the soil only lost 5%–8% of their mass, while sycamore leaves on the soil lost 2%–6% of their mass. On day 135, terrestrial leaves had still not decomposed as much as aquatic leaves had on day 28 (cottonwood leaves lost 21%–31% of their mass, and terrestrial sycamore leaves 5%–9% of their mass) (Fig. S2).

**Figure 2:**
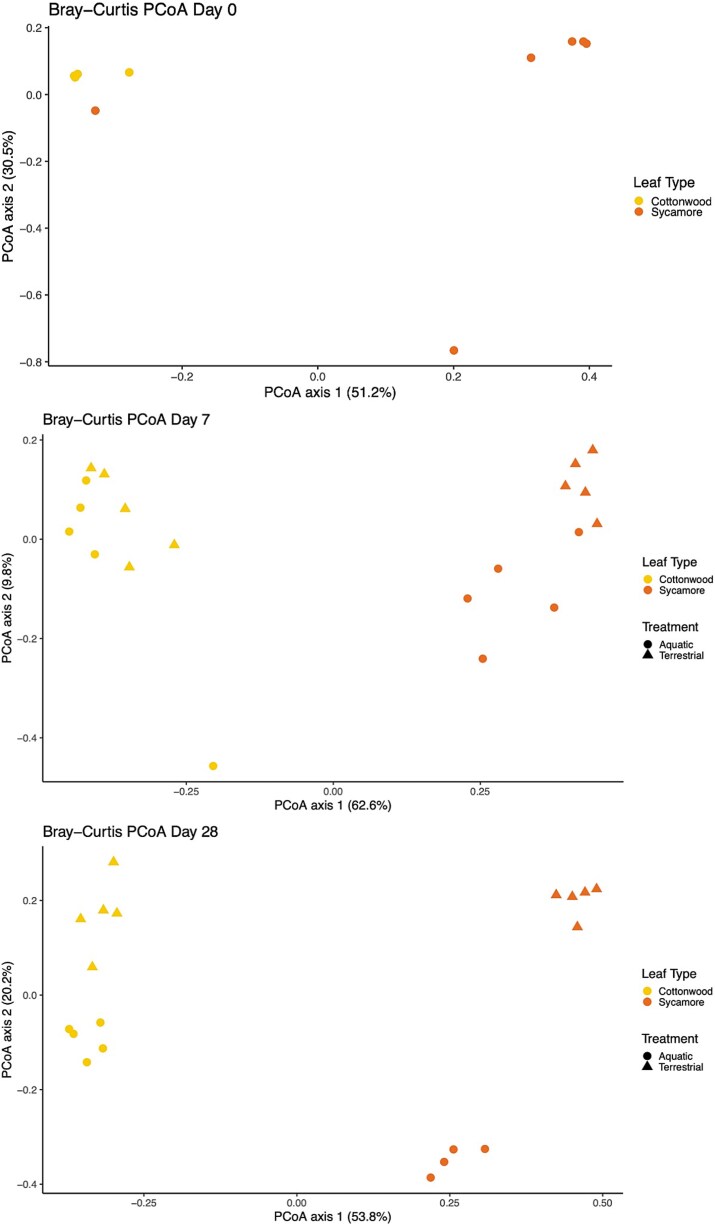
PCoA of Bray–Curtis dissimilarities among total fungal communities from litter packs across different treatments and leaf types. Each point represents one of five replicate litter packs in each combination of leaf type and treatment.

**Figure 3:**
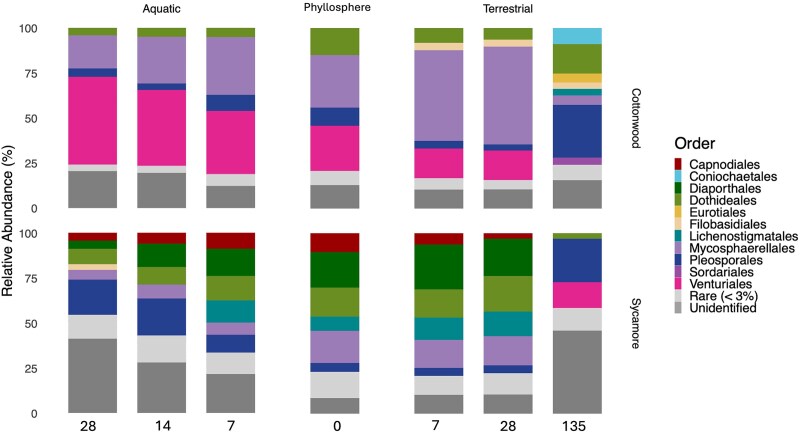
Relative abundances of fungal orders on leaves collected directly from phyllosphere, aquatic litter, and terrestrial litter. Leaves were taken from two tree species and collected from the aquatic and terrestrial environments at three time points.

We found that leaf type, environment (aquatic vs. terrestrial), and harvest date all significantly affected fungal community composition, but that leaf type was the strongest driver of community composition. Fungal communities differed significantly across leaf types, differences that began with initial phyllosphere communities and persisted throughout decomposition (Figs 2 and 3). Leaf type (cottonwood vs. sycamore), environment (aquatic vs. terrestrial), and harvest date significantly structured fungal assemblages during decomposition (Fig. 2) (PERMANOVA, *P* < .001). Leaf type was the strongest determinant, explaining 28% of the variance in community composition; harvest date explained 11% of the variance and environment explained 8% of the variance (Table S1). Before being placed in the stream or on the soil, cottonwood and sycamore leaves shared 24% of taxa in their phyllosphere communities (Fig. S3). After these leaves had been allowed to decompose in the stream or on the soil for 28 days, aquatic leaves shared 24% of actively growing taxa between leaf types (Fig. S3), while terrestrial leaves exhibited higher overlap, sharing 48% of growing taxa.

**Figure 4:**
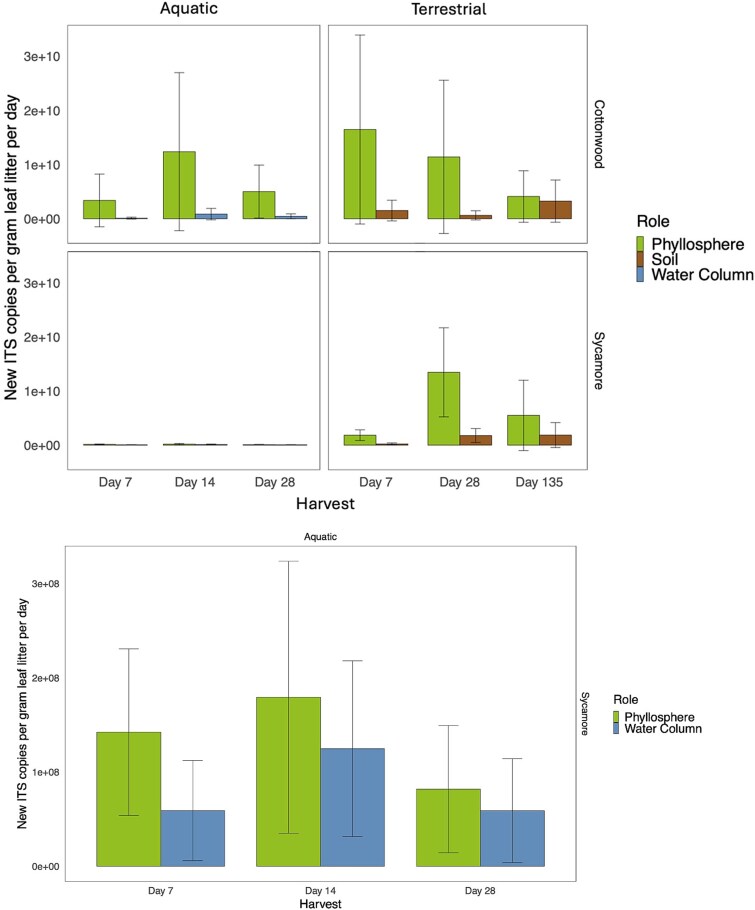
New ITS copies per gram of leaf litter per day for phyllosphere fungi and fungi colonizing from the soil or water column. This metric serves as a proxy for absolute growth rates. Error bars represent standard deviation. Panel (A) presents the full dataset, while panel (B) provides a magnified view of the aquatic sycamore subset.

Phyllosphere fungi made up the majority of both the fungal assemblages and the total new fungal growth on decomposing litter across litter types, environments, and harvest dates (Figs 1 and 4). Phyllosphere fungi accounted for 53%–97% of ITS gene copies in the river and 54%–93% on land (Fig. 1). As expected, the relative abundance of phyllosphere fungi was highest at earlier harvests (Fig. 1) and decreased significantly by the third harvest across all leaf types and environments (logistic regression, all *P* < .001). Fungi colonizing from the surrounding environment increased in abundance with time but never exceeded 50% of the total community. Phyllosphere fungi always contributed more new ITS copies per gram of leaf litter per day than fungi colonizing from the environment (Fig. 4).

**Figure 5:**
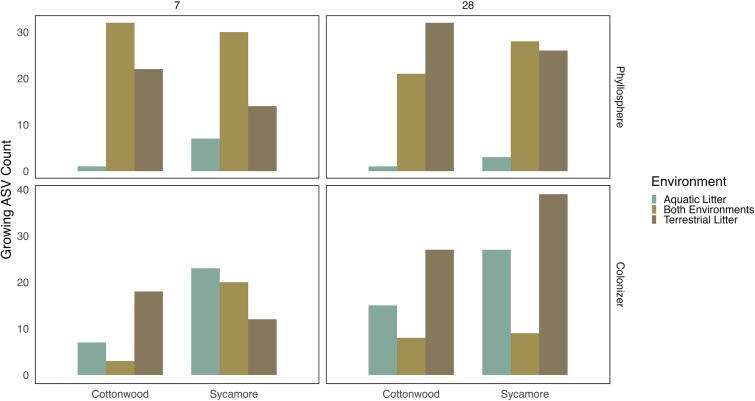
Number of growing taxa found on aquatic litter, terrestrial litter, or both. The top row represents phyllosphere fungi, while the bottom row represents fungi colonizing from the environment.

**Figure 6:**
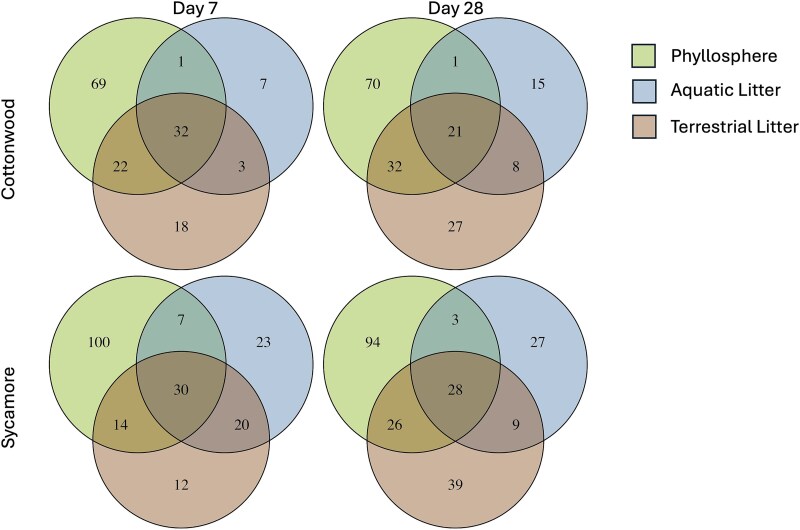
Growing fungal taxa shared among the phyllosphere, aquatic litter, and terrestrial litter on sycamore and cottonwood leaves at days 7 and 28.

Although aquatic and terrestrial environments exert very different selective pressures on microbial communities, many fungal taxa were growing across both environments. The majority of phyllosphere fungi with detectable growth rates on decomposing litter were growing in both environments (Figs 5 and 6). At day 7, 58%–59% of phyllosphere taxa growing on litter were growing in both environments. This pattern persisted at day 28; 39%–49% of phyllosphere taxa growing on litter were growing in both environments.

In contrast, the majority of colonizing fungi were specific to either aquatic or terrestrial environments (Fig. 5). On cottonwood litter, 65%–70% of growing taxa that colonized in the water were only found in aquatic litter packs and 77%–86% of growing taxa that colonized from soil were only found in terrestrial packs. Similarly, on sycamore litter, 53%–75% of taxa that colonized in the water were unique to the aquatic litter and 38%–81% that colonized from soil were only found in terrestrial packs. Some fungi colonized from both environments, especially on sycamore leaves at day 28 (36%).

Overall, absolute fungal growth was higher in terrestrial litter than in aquatic litter for both phyllosphere and colonizing taxa (Fig. 4). The majority (86%–98%) of growing phyllosphere fungi were growing in terrestrial litter, whereas only 41%–73% were growing in aquatic environments (Figs 5 and 6). Furthermore, just 2%–14% of phyllosphere fungi grew on aquatic litter alone, compared to 32%–60% growing on terrestrial litter alone. Additionally, terrestrial litter had higher richness, especially at day 135. Aquatic litter only had 33–80 growing ASVs, whereas terrestrial litter had 75–123 growing ASVs.

## Discussion

Contrary to our predictions, we found that many fungal taxa, particularly those that entered with the phyllosphere, were growing in both terrestrial and aquatic ecosystems. Given the strong abiotic differences between aquatic and terrestrial environments [15, 56], we expected greater divergence in fungal communities, as differing environmental factors selected for different fungal taxa [57]. The fact that many phyllosphere fungi were able to persist and grow in both environments suggests that those phyllosphere fungi are able to grow in multiple environments. In addition to shared phyllosphere fungi, some colonizing fungi were also growing in both environments. If these fungi truly colonized from the environment, rather than coming in with the leaf in quantities too small to detect, the cosmopolitan fungi support the idea of a riparian fungal meta-community rather than distinct soil vs. stream communities [58, 59].

While there were more growing fungal taxa shared between environments than expected, differences between aquatic and terrestrial fungal communities did increase over time in our study. The latest shared time point in our study was 28 days, which is about the minimum amount of time for fungal colonization of litter [60, 61]. It is possible that similarities between aquatic and terrestrial fungal communities in those 28 days reflect insufficient colonization time (although qSIP clearly demonstrated some fungal growth). Moreover, terrestrial litter was much less decomposed than aquatic litter, so while we can compare time points, we cannot compare decompositional stages (Fig. S2). Comparing aquatic and terrestrial communities at 50% mass loss instead of at fixed time points may reveal greater differences in fungal community structure.

Phyllosphere fungi dominate early decomposition but are typically overtaken by colonizing fungi on terrestrial litter by the fourth month or ~20% mass loss [9]. While we observed a decline in phyllosphere fungi over time, colonizing fungi never exceeded 50% relative abundance, even after 135 days (~4.5 months) of terrestrial decomposition. However, the treatments where phyllosphere fungi diminished the most—aquatic sycamore litter and terrestrial cottonwood litter—also had ~20% mass loss at their final time point, suggesting that mass loss may be a better predictor of phyllosphere abundance than number of days in the field. Faster decomposition strengthens priority effects [62], which may explain why colonizing fungi never established on rapidly decomposing aquatic cottonwood litter. In contrast, terrestrial sycamore litter had lost only 6% of its mass by day 135, suggesting minimal decomposition had occurred.

We found that leaf type was the most important factor for structuring fungal communities, more so than environment or time point. Leaf type can significantly impact fungal colonization rates, growth, and biomass during decomposition [63–65], with this impact usually attributed to leaf litter chemistry after leaf senescence. Yet initial phyllosphere community also differs between leaf types and also has a strong impact on litter decomposer communities [66, 67]. We posit that leaf litter chemistry matters even before leaf senescence by dictating which phyllosphere microbiome is recruited to the still living leaf. This phyllosphere community then dictates decomposer community; thus, the tree determines its own decomposition trajectory before the leaf even falls.

We found that phyllosphere fungi dominated both new fungal growth and relative abundance in aquatic and terrestrial environments. This is consistent with previous work on phyllosphere contributions to aquatic fungal activity [8, 68] and work on growth rates of initial fungi versus later colonizers on terrestrial litter [69]. While we did not quantify the role of phyllosphere in decomposition, phyllosphere fungi can accelerate aquatic and terrestrial litter breakdown [67, 70–72]. Since our study did not cover the whole process of terrestrial decomposition, further research is needed to determine whether phyllosphere fungi continue to be more active than colonizing fungi in later stages of terrestrial decomposition.

Using qSIP to calculate differences in absolute and relative growth rate, we found that fungi were, on average, growing faster on terrestrial litter and had higher richness on terrestrial litter. This was surprising given that shared fungi between aquatic and terrestrial environments grow more slowly on dry litter due to moisture limitation [73]. It is possible that the faster fungal growth on terrestrial litter we observed may reflect rewetting effects of adding water to dry leaf litter, which can inflate bacterial growth rates in soil by over 200% [74]. Additionally, since aquatic fungi allocate substantial biomass to spores [75], DNA extraction from leaf disks alone may have missed a portion of fungal biomass in the surrounding water. However, it is also possible that this difference in growth rates reflects a real ecological phenomenon, since fungal biomass and fungal richness are often higher on terrestrial litter than aquatic litter [15, 76–78]. Terrestrial decomposition happens at higher temperatures than aquatic decomposition, possibly leading to faster metabolic processes, and terrestrial litter leaches C much more slowly than aquatic litter, potentially providing more labile C compounds to terrestrial litter decomposers. Overall, there are very few studies comparing terrestrial and aquatic fungal growth, mostly in intermittent systems [42, 79]. Future work is needed to compare multiple fungal growth measurement methods at multiple riparian sites in order to establish whether faster terrestrial fungal growth can be generalized to other riparian systems.

Fungal leaf decomposition is a highly dynamic process, changing over time and depending on leaf and environmental factors. Using qSIP, we were able to demonstrate that leaf type was the strongest driver of fungal communities, regardless of time or environment, and that about a third of growing fungal taxa were shared between aquatic and terrestrial litter. Our results suggest that the trajectory of riparian decomposition is determined in the canopy, not on the forest floor or the stream bed. By selecting specific phyllosphere communities and defining leaf chemistry, trees exert a legacy effect that persists across ecosystem boundaries. Leaf type remained the primary driver of fungal community structure, overriding the stark abiotic differences between land and water. This indicates that to understand the fate of carbon in riparian zones, we must view the leaf and its microbiome as a unified package, one that functions coherently whether it falls on dry ground or drifts into a river.

## Supplementary Material

Supplementary_Information_Exp2_ycag187

## Data statement

The datasets generated during and/or analyzed during the current study are available in the SRA and Figshare repositories, https://figshare.com/s/aaafe23d50f5ce249b79 and BioProject:PRJNA1406271.
