# Supplementary material for: Fungi without borders: cross-ecosystem decomposition of riparian leaf litter
Source: ISME Commun. 2026 Jul 7;6(1):ycag187. doi: 10.1093/ismeco/ycag187 (PMC13398703; doi:10.1093/ismeco/ycag187)
Supplement: Supplementary_Information_Exp2_ycag187 [file supplementary_information_exp2_ycag187.docx]

**Fungi Without Borders: Cross-Ecosystem Decomposition of Riparian Leaf Litter**

Callie Ochs^1^, Michaela Hayer^1^, Raina Fitzpatrick^1^, Shannon Trottier^1^, Egbert Schwartz^1^, Bruce Hungate^1^, Jane Marks^1^

^1^Center for Ecosystem Science and Society, Department of Biology, Northern Arizona University, Flagstaff, AZ, USA

Appendix S1

Figure S1 – Mean relative growth rate (New ITS copies per total ITS copies per day) of phyllosphere and colonizing fungi.

Aquatic

Terrestrial

Harvest


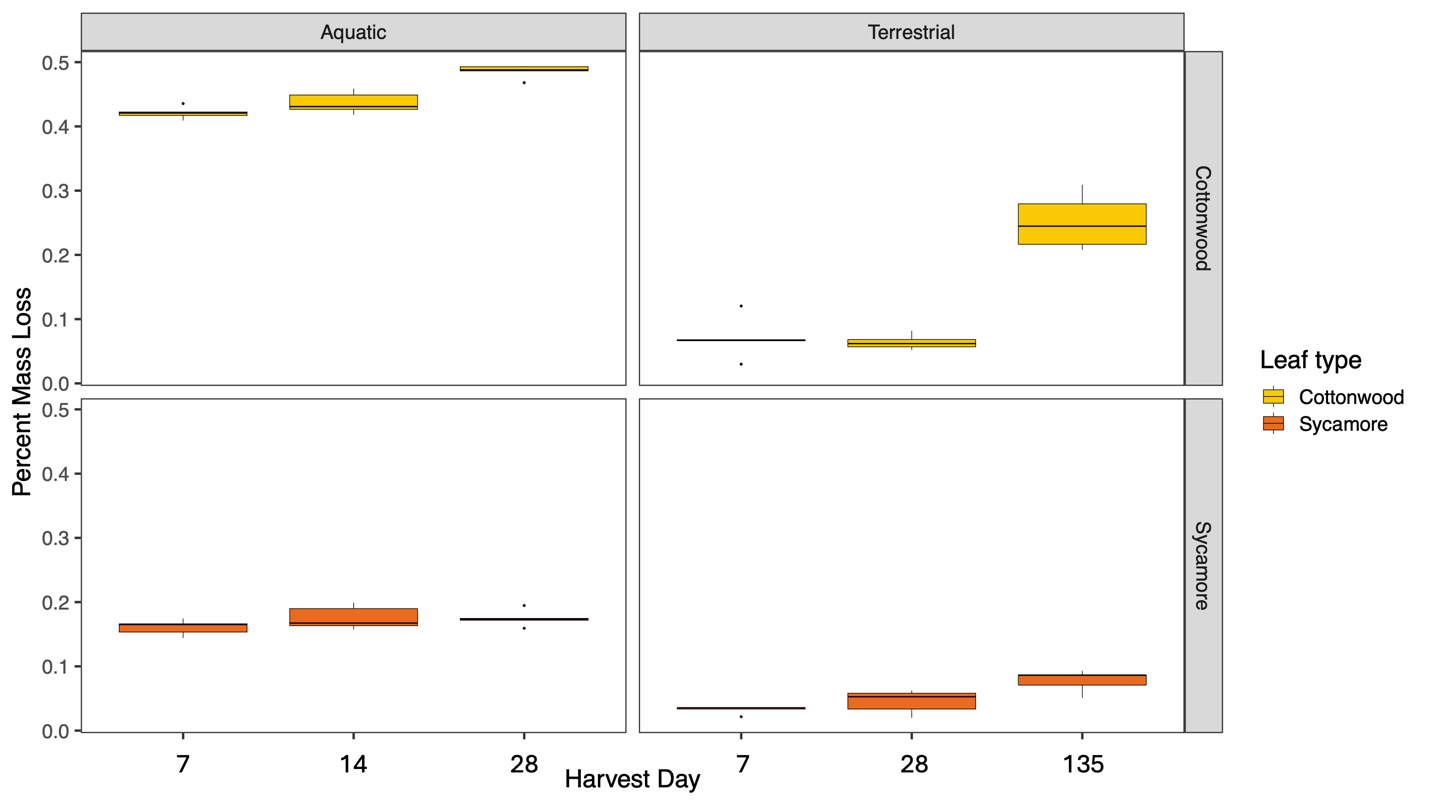


Figure S2 **–** Percent mass loss of Fremont cottonwood (*Populus fremontii*) and Arizona sycamore (*Platanus wrightii*) leaf litter in aquatic and terrestrial environments across three time points. Sampling intervals for the aquatic environment were 7, 14, and 28 days, whereas those for the terrestrial environment were 7, 28, and 135 days.


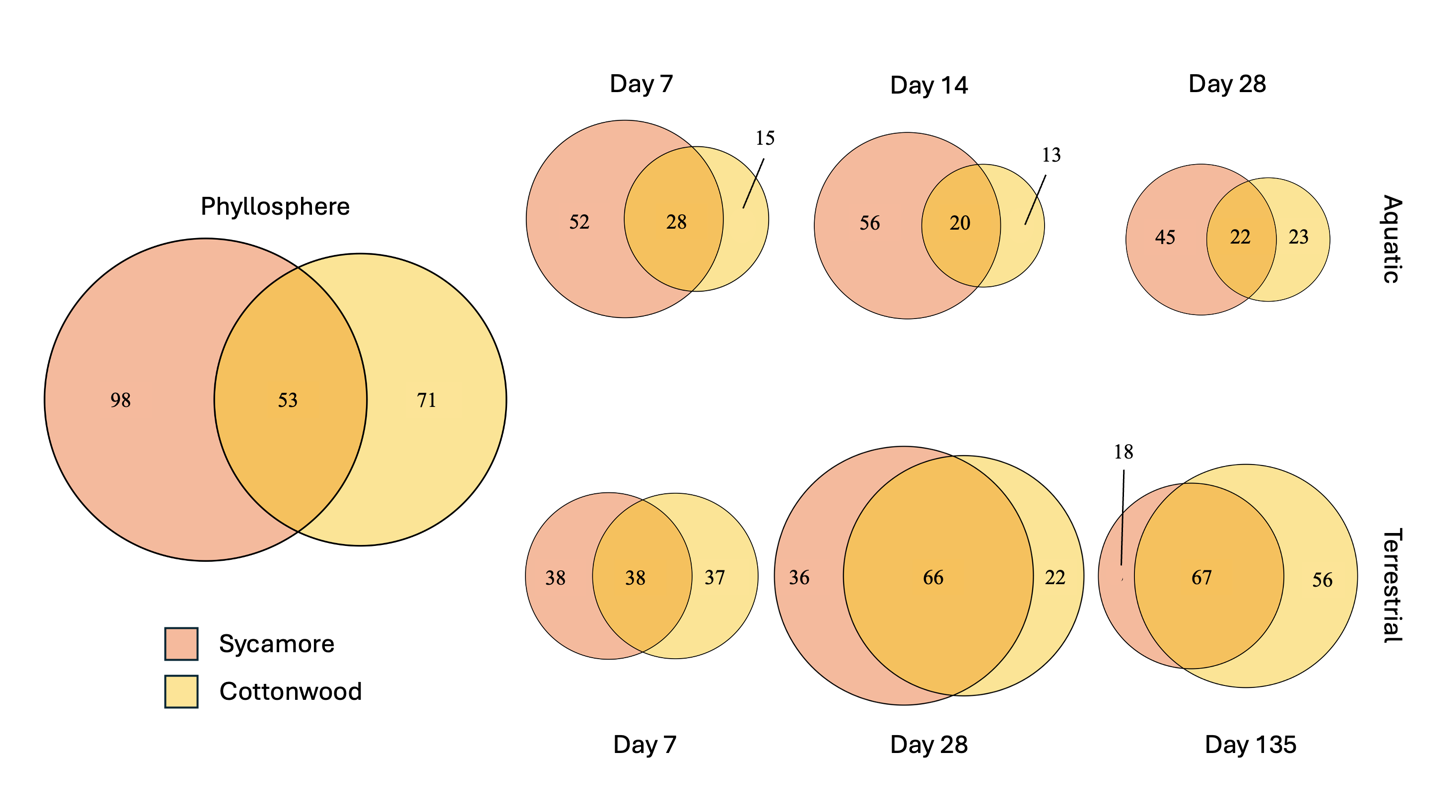
Figure S3 – Active fungal taxa shared between sycamore and cottonwood litter in aquatic and terrestrial environments across three time points.

Figure S3 – Mean relative growth rate (New ITS copies per total ITS copies per day) of phyllosphere and colonizing fungi.

Aquatic

Terrestrial

Harvest

Table S1 – PERMANOVA statistics

|  | **df** | **SumofSquares** | **R2** | **F** | **Pr(>F)** |
| --- | --- | --- | --- | --- | --- |
| Leaf_type | 1 | 4.7465828 | 0.275959624 | 51.953840 | 0.0001 |
| Treatment | 2 | 1.4365387 | 0.083518332 | 7.861835 | 0.0001 |
| Harvest | 3 | 1.8871915 | 0.109718648 | 6.885434 | 0.0001 |
| Leaf_type:Treatment | 2 | 1.2096088 | 0.070324949 | 6.619902 | 0.0001 |
| Leaf_type:Harvest | 3 | 2.6129542 | 0.151913467 | 9.533385 | 0.0001 |
| Treatment:Harvest | 1 | 0.1586302 | 0.009222534 | 1.736290 | 0.1181 |
| Leaf_type:Treatment:Harvest | 1 | 0.1238893 | 0.007202748 | 1.356033 | 0.1975 |
| Residual | 55 | 5.0248847 | 0.292139699 | *NA* | *NA* |
| Total | 68 | 17.2002802 | 1.000000000 | *NA* | *NA* |

Table S2 – Five most abundant ASVs in each treatment category. Taxon is listed down to the finest taxonomic assignment per ASV.

| Treatment | Taxon (finest taxonomic resolution) | Relative abundance (%) |
| --- | --- | --- |
| Cottonwood Terrestrial 7 | f__Mycosphaerellaceae | 50.1 |
| Cottonwood Terrestrial 7 | f__Venturiaceae | 16.4 |
| Cottonwood Terrestrial 7 | p__Ascomycota | 9.2 |
| Cottonwood Terrestrial 7 | Aureobasidium pullulans | 7.9 |
| Cottonwood Terrestrial 7 | Filobasidium chernovii | 2.9 |
| Cottonwood Terrestrial 28 | f__Mycosphaerellaceae | 54.3 |
| Cottonwood Terrestrial 28 | f__Venturiaceae | 16.3 |
| Cottonwood Terrestrial 28 | p__Ascomycota | 9.8 |
| Cottonwood Terrestrial 28 | Aureobasidium pullulans | 6.1 |
| Cottonwood Terrestrial 28 | Filobasidium chernovii | 2.2 |
| Cottonwood Terrestrial 135 | g__Alternaria | 21 |
| Cottonwood Terrestrial 135 | p__Ascomycota | 14 |
| Cottonwood Terrestrial 135 | Aureobasidium pullulans | 13.7 |
| Cottonwood Terrestrial 135 | g__Coniochaeta | 6.8 |
| Cottonwood Terrestrial 135 | f__Mycosphaerellaceae | 4.9 |
| Cottonwood Aquatic 7 | f__Venturiaceae | 34.8 |
| Cottonwood Aquatic 7 | f__Mycosphaerellaceae | 32.1 |
| Cottonwood Aquatic 7 | p__Ascomycota | 11.7 |
| Cottonwood Aquatic 7 | g__Alternaria | 6.8 |
| Cottonwood Aquatic 7 | Aureobasidium pullulans | 4 |
| Cottonwood Aquatic 14 | f__Venturiaceae | 42 |
| Cottonwood Aquatic 14 | f__Mycosphaerellaceae | 25.9 |
| Cottonwood Aquatic 14 | p__Ascomycota | 17.8 |
| Cottonwood Aquatic 14 | Aureobasidium pullulans | 4.4 |
| Cottonwood Aquatic 14 | g__Alternaria | 2 |
| Cottonwood Aquatic 28 | f__Venturiaceae | 48.5 |
| Cottonwood Aquatic 28 | p__Ascomycota | 18.9 |
| Cottonwood Aquatic 28 | f__Mycosphaerellaceae | 18.4 |
| Cottonwood Aquatic 28 | Aureobasidium pullulans | 4.1 |
| Cottonwood Aquatic 28 | g__Alternaria | 3.7 |
| Sycamore Terrestrial 7 | f__Gnomoniaceae | 24.9 |
| Sycamore Terrestrial 7 | f__Mycosphaerellaceae | 15.2 |
| Sycamore Terrestrial 7 | Aureobasidium pullulans | 12.7 |
| Sycamore Terrestrial 7 | g__Phaeococcomyces | 11.6 |
| Sycamore Terrestrial 7 | f__Cladoporiaceae | 6 |
| Sycamore Terrestrial 28 | f__Gnomoniaceae | 20.7 |
| Sycamore Terrestrial 28 | Aureobasidium pullulans | 16.9 |
| Sycamore Terrestrial 28 | f__Mycosphaerellaceae | 16.1 |
| Sycamore Terrestrial 28 | g__Phaeococcomyces | 10.5 |
| Sycamore Terrestrial 28 | c__Microbotryomycetes | 3.9 |
| Sycamore Terrestrial 135 | p__Ascomycota | 40.2 |
| Cottonwood Phyllosphere | f__Mycosphaerellaceae | 29.1 |
| Cottonwood Phyllosphere | f__Venturiaceae | 25 |
| Cottonwood Phyllosphere | Aureobasidium pullulans | 14.7 |
| Cottonwood Phyllosphere | p__Ascomycota | 11.2 |
| Cottonwood Phyllosphere | g__Alternaria | 7.9 |
| Sycamore Phyllosphere | f__Gnomoniaceae | 19.3 |
| Sycamore Phyllosphere | f__Mycosphaerellaceae | 16.5 |
| Sycamore Phyllosphere | Aureobasidium pullulans | 15 |
| Sycamore Phyllosphere | o__Capnodiales | 9.4 |
| Sycamore Phyllosphere | p__Ascomycota | 5.9 |
